# Comparative transcriptomics analysis of developing peanut (*Arachis hypogaea* L.) pods reveals candidate genes affecting peanut seed size

**DOI:** 10.3389/fpls.2022.958808

**Published:** 2022-09-12

**Authors:** Yue Wu, Ziqi Sun, Feiyan Qi, Mengdi Tian, Juan Wang, Ruifang Zhao, Xiao Wang, Xiaohui Wu, Xinlong Shi, Hongfei Liu, Wenzhao Dong, Bingyan Huang, Zheng Zheng, Xinyou Zhang

**Affiliations:** ^1^School of Life Sciences, Zhengzhou University, Zhengzhou, Henan, China; ^2^Henan Academy of Agricultural Sciences, Henan Academy of Crop Molecular Breeding, State Industrial Innovation Center of Biological Breeding, Key Laboratory of Oil Crops in Huang-Huai-Hai Plains, Ministry of Agriculture, Henan Provincial Key Laboratory for Oil Crops Improvement, Innovation Base of Zhengzhou University, Zhengzhou, Henan, China; ^3^College of Agronomy, Henan Agricultural University, Zhengzhou, Henan, China; ^4^College of Agriculture, Henan University of Science and Technology, Luoyang, China; ^5^School of Agricultural Sciences, Zhengzhou University, Zhengzhou, Henan, China

**Keywords:** peanut, phytohormone, MAPK signaling pathway, transcription factor, pod development, pod size

## Abstract

Pod size is one of the most important agronomic features of peanuts, which directly affects peanut yield. Studies on the regulation mechanism underpinning pod size in cultivated peanuts remain hitherto limited compared to model plant systems. To better understand the molecular elements that underpin peanut pod development, we conducted a comprehensive analysis of chronological transcriptomics during pod development in four peanut accessions with similar genetic backgrounds, but varying pod sizes. Several plant transcription factors, phytohormones, and the mitogen-activated protein kinase (MAPK) signaling pathways were significantly enriched among differentially expressed genes (DEGs) at five consecutive developmental stages, revealing an eclectic range of candidate genes, including *PNC*, *YUC*, and *IAA* that regulate auxin synthesis and metabolism, *CYCD* and *CYCU* that regulate cell differentiation and proliferation, and *GASA* that regulates seed size and pod elongation *via* gibberellin pathway. It is plausible that *MPK3* promotes integument cell division and regulates mitotic activity through phosphorylation, and the interactions between these genes form a network of molecular pathways that affect peanut pod size. Furthermore, two variant sites, *GCP4* and *RPPL1*, were identified which are stable at the QTL interval for seed size attributes and function in plant cell tissue microtubule nucleation. These findings may facilitate the identification of candidate genes that regulate pod size and impart yield improvement in cultivated peanuts.

## Introduction

Cultivated peanut (*Arachis hypogaea* L.) is one of the most important cash crops, providing vegetable oil and proteins to consumers in many parts of the world (Zheng et al., 2018). In 2020, the global peanut acreage reached 32 million hectares, producing about 54 million metric tons, of which China produced the most, more than 18 million metric tons.^1^ Because of the limitation of arable lands and the competition from other food crops, genetic improvement of peanut yield per unit area has long been a target of priority sought after by peanut breeders worldwide in order to meet the growing demands for peanuts worldwide.

As a key component in peanut yield, the peanut pod is composed of seed and shell. The peanut shell is developed from the ovary wall, which protects peanut seeds from biotic and abiotic stresses and continuously provides nutrients for seed development (Bruce et al., 1975). Large shells also provide more environmental space and nutrients for seeds, which are considered a favorable trait for yield improvement (Zhou et al., 2022). The seed develops from double fertilization and seed size is determined when the endosperm cellularization is completed (Garcia et al., 2003). At the cellular level, seed size depends on the synergism of cell proliferation and cell expansion during growth and development (Hepworth and Lenhard, 2014). Pod size is recognized as a quantitative trait that is regulated by multiple genes (Mizukami and Fischer, 2000). Peanut has made progress in molecular markers, genetic map construction, and quantitative trait loci (QTL) localization studies for seed size-related traits in recent years (Wang et al., 2018; Mondal and Badigannavar, 2019; Zhang S. et al., 2019). The major QTLs for seed length were mainly located on chromosomes A2, A5, B6, and B7, the major QTLs associated with seed width were located on chromosomes B6 and B7, and the major QTLs identified for 100-seed weight were located on chromosomes B6 and B7. These major QTLs not only regulated seed size but also regulated pod size. Several stable major QTLs for pod size in peanuts have been identified by leveraging QTL mapping (Wang et al., 2018; Zhang S. et al., 2019), but the physical intervals of these QTLs are too large to be practically useful due to the complexity of the peanut genome structure (Bertioli et al., 2019; Chen et al., 2019; Zhuang et al., 2019). Spatiotemporal dynamic transcriptome analysis, nevertheless, may facilitate the investigation into the molecular mechanisms that underpin seed development and ultimately determine pod/seed size.

Numerous genes have been discovered to regulate pod size in legumes. The phytohormone pathway plays a significant role in the regulation of pod size (Li and Li, 2016; Li et al., 2019). For instance, the *PsTAR2* encodes an aminotransferase that is involved in the growth hormone production route and catalyzes the conversion of tryptophan to IPyA. The *tar2-1* mutation in *Pisum sativum* led to smaller seeds, lower starch content, and crumpled seeds (McAdam et al., 2017). Overexpression of *GA20ox* dramatically enhanced seed size, the gene was firmly connected to a localized seed weight QTL, and expression of *GA20ox* was much higher in cultivated soybean (*Glycine max*) than in wild soybean (*Glycine soja*) (Lu et al., 2016). Several plant transcription factors (TF) play imperative roles in plant seed development (Alizadeh et al., 2021; Su et al., 2021). For example, WRKY36 suppresses grain enlargement by attenuating gibberellin signaling in rice (Lan et al., 2020), and MYB56 regulates seed size by modulating cell development in Arabidopsis (Zhang et al., 2013). In addition, several signaling pathways, such as the MAPK signaling pathway (Xu et al., 2018a; Liu et al., 2021; Tian et al., 2021), ubiquitin-proteasome pathway (Li and Li, 2014; Linden and Callis, 2020), and phytohormone-mediated pathways (Li et al., 2019; Qadir et al., 2021), have been found to regulate seed size in both rice and Arabidopsis that are the model plants for monocotyledonous and dicotyledonous plants, respectively. In rice, OsMKKK10, OsMKK4, and OsMAPK6 act as a cascade to regulate grain size, and the loss-of-function mutant of *OsMAPK6* was found to produce markedly smaller than normal grains due to reduced cell proliferation in a spikelet (Duan et al., 2014; Liu et al., 2015; Xu et al., 2018b).

In this study, the developing pods of two peanut varieties with contrasting pod sizes, together with those of their derivative recombinant inbred lines (RIL), were used for transcriptome analysis. The in-depth transcriptome dynamics and transcriptional network that are associated with pod development were explored, in order to identify the molecular regulatory elements and functional genes that are differentially expressed between the genotypes with large or small pod sizes during seed development. The overlapping of known QTLs and the differentially expressed genes (DEGs) with respect to pod size and the identification of single nucleotide polymorphisms (SNPs) allowed for the selection of candidate genes that regulate pod size. This study may shed more light on the underlying molecular mechanisms of peanut pod development, providing a set of pod size-associated key genes that could be explored and utilized in future breeding programs and genetic modifications for peanut yield improvement.

## Materials and methods

### Plant materials and sampling

A RIL population (F_12_) of 329 lines was derived from a cross between YH15 (female parent) and W1202 (male parent) by single-seed descent (SSD) (Sun et al., 2022). YH15 is a large pod peanut cultivar from the Henan Academy of Agricultural Science, and W1202 is a breeding line with a relatively small pod. The largest pod line of S181523 and the smallest pod line of S181517 were selected from the RIL population. YH15, W1202, together with S181523 and S181517 were planted in the experimental fields in Zhengzhou, Henan Province, China in the summer of 2020. Flowers were color-tagged and the pods of 25 days (d) after flowering (DAF) were sampled. At both 35 and 45 DAF, shells and seeds were separately sampled. The sampling time at 25, 35, and 45 DAF corresponds to the early, middle, and late developmental stages, respectively. The five samples are referred to as Pod-25, Shell-35, Seed-35, Shell-45, and Seed-45, each of which was repeated three times and immediately frozen in liquid nitrogen and stored at −80°C until further use. In order to avoid the influence of environmental factors, YH15, W1202, S181523, and S181517 were planted at three locations: Zhengzhou (ZZ, Henan Province, China), Shangqiu (SQ, Henan Province, China), and Weifang (WF, Shandong Province, China). The hundred seed weight (HSW) and the length and width of pod and seed for the four genotypes were measured at maturity in these three environments.

### Anatomical observation of seed and shell

In this study, the cell developmental morphology of seed-35, seed-45, shell-35, and shell-45 developmental stages in YH15, W1202, S181523, and S181517 were observed. The plant samples were first fixed in FAA fixative, dehydrated, and embedded in paraffin. Then they were dewaxed, treated with toluidine blue, and sealed with neutral gum. Finally, the process was observed under an optical microscope, and the morphological characteristics of each period were recorded and photographed. The image analysis software ImageJ was used to quantify and graphed in GraphPad Prism 8. The significance level was set to *P* < 0.05.

### RNA extraction, library construction, and deep-sequencing

Total RNA was extracted with TaKaRa MiniBEST Plant RNA Extraction Kit (Takara Bio Inc., Kusatsu, Shiga, Japan). RNA quality was checked using a 2100 Bioanalyzer (Agilent Technologies, Santa Clara, CA, United States) to ensure all the RNA samples meet the following criteria: OD260/OD280 ≥ 1.8, the 28S/18S ratio closer to 2. RNA integrity was determined by 1.5% agarose gel electrophoresis. The high quality RNA samples were then fractionated, purified, screened, and finally used to construct cDNA libraries for sequencing on the Illumina Hiseq platform in Beijing Genome Institute (BGI, Shenzhen, China). The RNA-seq raw data have been submitted to the NCBI database (BioProject with the SRA accession number: PRJNA847769).

### Read mapping and single nucleotide polymorphism calling

Trimmomatic v0.39 (Bolger et al., 2014) was used to remove adapters, reads with more than 5% unknown bases (N content), and low-quality reads from raw data. Fastqc^2^ was used to evaluate the quality of clean data and then the statistics were compiled into a summary by Multiqc (Ewels et al., 2016). All clean reads were aligned to the reference genome of *Arachis hypogaea* cv. Tifrunner^3^ using HISAT2 (Zhang et al., 2021).

Samtools (Li, 2011) was used to convert sam files into bam files that were then sorted to create an index and the sequencing depth was counted. The outputs from sequence alignment were processed by featureCounts (Liao et al., 2019) to get count values for all the genes, which were then transformed into FPKM/TPM (Fragments Per Kilobase Million/Transcripts Per Kilobase Million) for subsequent analysis using a built R script (R Core Team, 2021, R Foundation for Statistical Computing). The log10 ratio approach was used to quantify relative gene expression levels among all the samples, and Pearson’s correlation coefficients were calculated between every two samples. Principal component analysis (PCA) was conducted and the results were plotted using the Omicshare online tool,^4^ and the sample correlation heatmaps were plotted against the sample relative gene expression level using the R package function ggplot2 and GGally (Ginestet, 2011).

The Picard-tools^5^ and Samtools were used to sort and highlight duplicate readings and reorder the alignment results for each sample. The Genome Analysis Toolkit GATK (Poplin et al., 2018) was used to call insertions and deletions (Indels) and SNPs with the following filter parameters: Quality by Depth (QD) below 5 and Quality Score (QUAL) below 30, -window 35, and -cluster 3. The SnpEff (Cingolani et al., 2014) variant annotation tool implemented in GATK was used to estimate the nucleotide sequence variability of their mRNAs on a gene-by-gene basis. R package function maftools with visualization capabilities (Mayakonda et al., 2018) were used to assess mutations and map mutation spectrum characteristics.

### Gene annotation

Functional annotation of transcriptome data was carried out using the databases of NR (NCBI non-redundant protein sequences), Swissprot (Manually annotated and reviewed protein sequences), KEGG (Kyoto Encyclopedia of Genes and Genomes), and GO (Gene Ontology). For TF annotation, EMBOSS function getorf was used to find gene open reading frames (ORFs) that were aligned to identify the TF protein domains using Hmmsearch (Potter et al., 2018), and finally annotated based on TF family properties using PlantTFdb^6^ (Jin et al., 2013). The *E*-value for all BLAST searches was set to 1e^–5^.

### Differential gene expression analyses and enrichment analysis

The DEGseq2 (Love et al., 2014) method was used to identify DEGs, and the screening criteria were set as *P*-value < 0.05 and | log2 ^(foldchange)^ | > 1. The DEGs of YH15 vs. W1202 and S181523 vs. S181517 at each developmental stage were taken to intersect and recognized as the final DEGs since S181523 and S181517 share the same genetic background as their parents YH15 and W1202. The DEGs were functionally identified based on the GO/KEGG annotation and categorization, and the Phyper function of R software^7^ was used for enrichment analysis to determine *P*-value, which was subsequently corrected by the False Discovery Rate (FDR). Significant enrichment was defined as functional when *P*-value was less than 0.05. STRING (Szklarczyk et al., 2021) was used to uncover detailed interconnections amongst DEG lists. The DEG protein-protein interaction (PPI) network was visualized using the Cytoscape program (Shannon et al., 2003).

### Quantitative real-time PCR validation for differentially expressed genes

To further validate the reliability of the DEGs obtained by RNA-Seq analysis, the expression levels of several selected genes were measured by quantitative real-time reverse transcriptase polymerase chain reaction (qRT-PCR) using the reserved RNA samples. First, RNA was reverse transcribed to 1st strand cDNA using the PrimeScript™ II 1st Strand cDNA Synthesis Kit (TaKaRa). Primers were designed using Oligo7 Primer Analysis Software (Molecular Biology Insights Inc, Colorado Springs, CO, United States) and their gene specificity was evaluated using Primer-BLAST.^8^ Finally, qRT-PCR analysis was performed using Power Up™ SYBR™ Green Master Mix (Applied Biology Inc., Irvine, CA, United States). *ADH3* was chosen as a reference gene and at least three technical replicates were performed for all the genes in each pool (Brand and Hovav, 2010). Differences in gene expression between the two samples were calculated by the 2^–ΔΔCt^ method (Livak and Schmittgen, 2001) and correlation analysis was performed using GraphPad Prism 8 (GraphPad Software, La Jolla, CA, United States) to assess the concordance between RT-qPCR results and RNA-seq data.

## Results

### Phenotyping for four peanut materials

**FIGURE 1:**
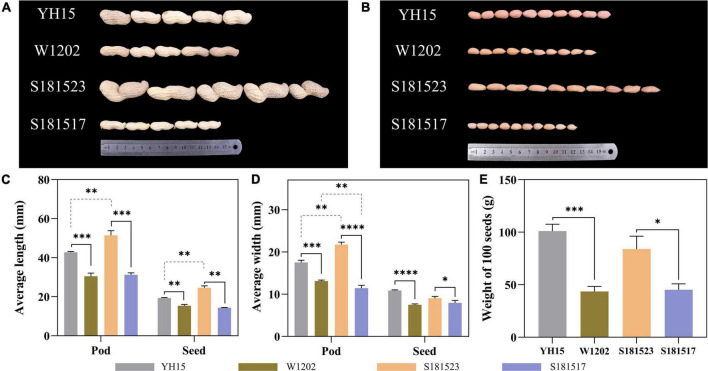
Phenotypes and statistics of YH15, W1202, S181523, and S181527. **(A)** Pods of YH15, W1202, S181523, and S181527. **(B)** Seeds of YH15, W1202, S181523, and S181527. **(C–E)** Statistics of pod and seed length, width, and 100-seed weight. Error bars represent SD. *, **, ***, and **** represent *p* ≤ 0.05, **p ≤ 0.01, ***p ≤ 0.001, and ****p ≤ 0.0001, respectively.

**FIGURE 2:**
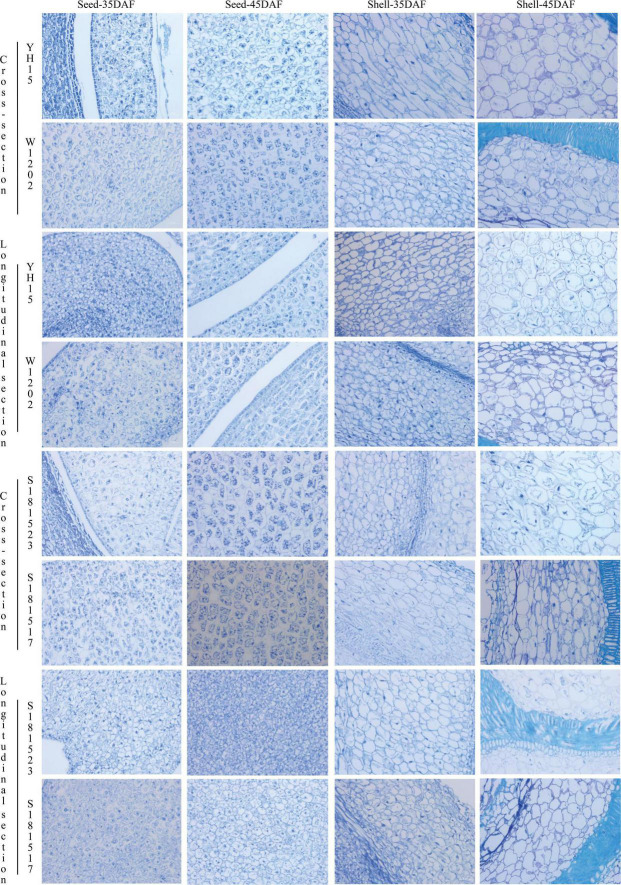
Paraffin sections of peanut seed and shell. All magnifications are ×20. 20× Bar = 10 μm.

Significant variations in the pod sizes and seed sizes were found between S181523 and S181517, and between their parents, YH15 and W1202 as well. As shown in Figure 1, the pod sizes of YH15 were significantly higher than W1202, similarly, S181523 was significantly higher than S181517. By analyzing the planting results in ZZ, SQ, and WF, it was found that the average pod length, average seed length, average pod width, average seed width, and 100-seed weight of the large-grained genotypes were significantly higher than those of the small-grained genotypes, which were among parents, pedigree, respectively (Supplementary Table 1). The largest pod pedigree of S181523 showed superior phenotypic traits to YH15 (a large pod of the female parent) in terms of pod length, seed length, and pod width. The smallest pod pedigree of S181517 showed superior phenotypic traits to W1202 (a small pod of a male parent) only in terms of seed length and pod width. Observation of the seed cross-section revealed that there was no significant change in cell size between large and small grain peanuts, but there was a significant increase in cell size and closer intercellular arrangement on 45 DAF compared to 35 DAF (Figure 2), such as the cell area of S181523 seed was significantly larger (Supplementary Figure 1A). The number of cells in the longitudinal-section of the seed was significantly higher in the large-grain peanuts. For example, S181523 cells were significantly more numerous than S181517 at 45 DAF (Supplementary Figure 1B). The inclusions gradually increased during the development. On the other hand, the shell staining results showed that more cells matured, nuclei disappeared, and increased cell area at 45 DAF than 35 DAF (Supplementary Figure 1C).

### Transcriptome profile of seed development

A total of 2.29 billion high-quality clean reads with Q20 ≥ 96.04%, GC ≥ 43.50% were generated for all four genotypes, including YH15, W1202, S181523, and S181517, with the mapping rate of 76.57∼98.43% (Supplementary Table 2). Due to the low mapping rate (51.93%) of the YH15_SHELL35_2, it was removed for further analysis.

A total of 67,005 expressed genes were detected, which gradually declined as the seed developed toward maturity. For example, in the pod tissue differentiation stage (Pod-25), the expressed genes were significantly more abundant than in the middle and late pod maturation stages. However, there was no discernible variation in the number of expressed genes between genotypes at each developmental stage (Supplementary Figure 2). PCA, correlation analysis, and expression distribution analysis showed high correlations among similar tissues/developmental stages, and the distribution of gene expression levels in the samples was uniform, manifesting data validity (Supplementary Figures 3–5). Therefore, the average FPKM value of the three replicates was calculated as the gene expression level of each sample. These high-density time series transcripts could be classified into five groups based on developmental stages: Shell-35, Shell-45, Pod-25, Seed-35, and Seed-45 following PCA analysis (Supplementary Figure 3).

### Functional annotation

For functional annotation, all the sequences were blasted with the sequences in Nr, Swissprot, Eggnog, GO, and KEGG public databases. As a result, a total of 21,919 genes were commonly annotated by all the five above databases, accounting for 32.67% of the expressed genes, and 66,465 genes were annotated in at least one database, accounting for 99.07% of the expressed genes (Supplementary Figure 6). Genes annotated in Nr and Swissprot were 65,603 and 50,591, respectively. The 27,330 genes annotated in GO were divided into three components, 41.01% in biological processes, 25.17% in cell composition, and 33.82% in molecular functions.

### Differentially expressed gene identification and enrichment analysis

**FIGURE 3:**
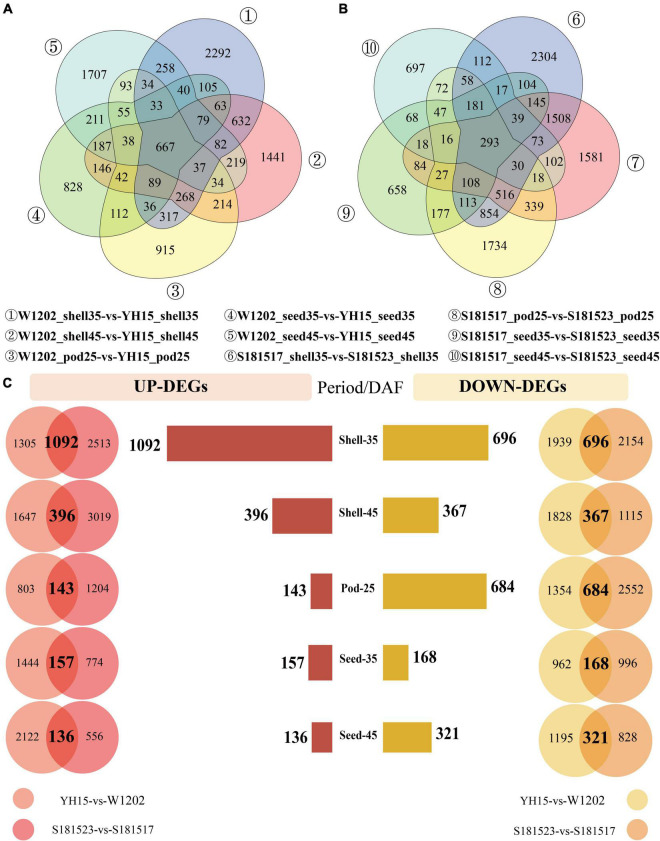
Analysis of differentially expressed genes (DEGs) in the comparisons of YH15 vs. W1202 and S181523 vs. S181527. (| log2 fold-change| > 1, corrected *p*-value < 0.05). **(A)** The number of DEGs between YH15 and W1202 at each pod developmental stage. **(B)** The number of DEGs between S181523 and S181527 at each pod developmental stage. **(C)** The number of intersected DEG s in five phases: Shell-35, Shell-45, Pod-25, Seed-35, and Seed-45 DAF.

To identify candidate genes implicated in pod size, DEGs were analyzed between YH15 and W1202, and S181523 and S181517 at five developmental stages including Pod-25, Shell-35, Seed-35, Shell-45, and Seed-45. For YH15 and W1202, a total of 16,875 DEGs were identified at five pod developmental stages, including 5,032 DEGs at the Shell-35 stage, followed by 4,238, 3,774, 2,986, and 2,731, in Shell-45, Seed-45, Pod-25, and Seed-35, respectively (Figure 3A and Supplementary Table 3). The highest number of DEG between S181523 and S181517 was 6,455 in Shell-35, followed by 4,897, 4,583, 2,095, and 1,841 in Shell-45, Pod-25, Seed-35, and Seed-45 (Figure 3B and Supplementary Table 3). Interestingly, there were 219 DEGs were commonly identified in all the comparisons (Supplementary Figure 7). In order to eliminate the interference caused by background noise during the analysis and target the most important genes with pod size traits in the DEGs, the DEGs obtained from the comparison of S181523 and S181517 were intersected with the DEGs obtained from the comparison of YH15 and W1202 at the same developmental stage (Figure 3C). Similarly, in the comparison of large-grained genotype and small-grained genotype, the highest number of DEGs were identified in Shell-35 period with 1,092 and 696 genes being upregulated and downregulated, respectively (Figure 3C). The intersected DEGs were used for further analysis.

**FIGURE 4:**
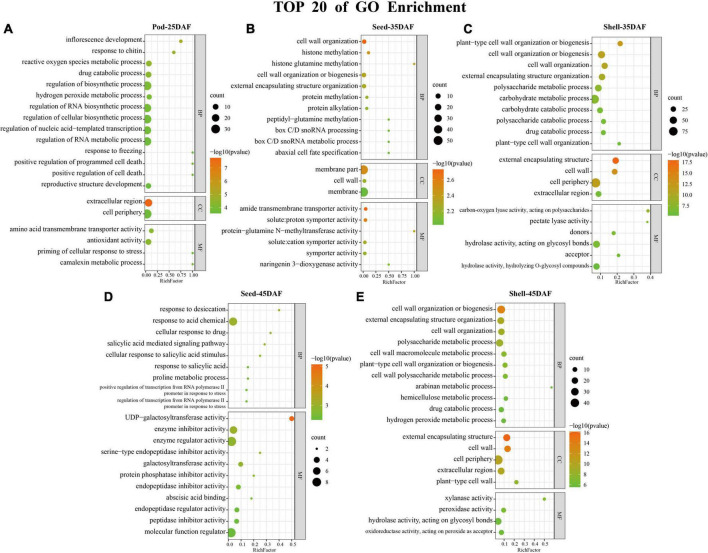
Gene Ontology (GO) Enrichment analysis. **(A–E)** Top 20 terms of GO enrichment results in five phases: Pod-25, Seed-35, Shell-35, Seed-45, and Shell-45 DAF. The color scale indicates significance (corrected *p*-value). The size of the circle represents the number of genes enriched by that term.

Gene Ontology enrichment analysis was performed on the intersected DEGs of 1,788, 763, 827, 325, and 457 in Shell-35, Shell-45, Pod-25, Seed-35, and Seed-45, respectively (Figure 4 and Supplementary Table 4). During the Pod-25 period, inflorescence development (GO:0010229), extracellular region (GO:0005576) and transmembrane transporter activity (GO:0015171) were most significantly enriched in biological process (BP), cellular component (CC) and molecular function (MF) (Figure 4A). During the Seed-35 period, cell wall organization (GO:0071555), membrane par (GO:0044425) and transmembrane transporter activity (GO:0042887) were most significantly enriched in BP, CC, and MF (Figure 4B). During the Shell-35 period, DEGs were most enriched in carbohydrate metabolic process (GO:0005975) and catabolic process (GO:0009056). Cell cycle-related terms were also significantly enriched, such as positive regulation of cell proliferation (GO:0008284), cell cycle (GO:0007049), and mitotic cell cycle process (GO:1903047) (Figure 4C). During the Seed-45 period, response to desiccation (GO:0009269), extracellular region (GO:0005576) and UDP-galactosyltransferase activity (GO:0035250) were most significantly enriched in BP and MF (Figure 4D). Finally, cell wall organization or biogenesis (GO:0071554) and external encapsulating structure (GO:0030312) were most significantly enriched in BP and CC during the Shell-45 period, respectively (Figure 4E).

**FIGURE 5:**
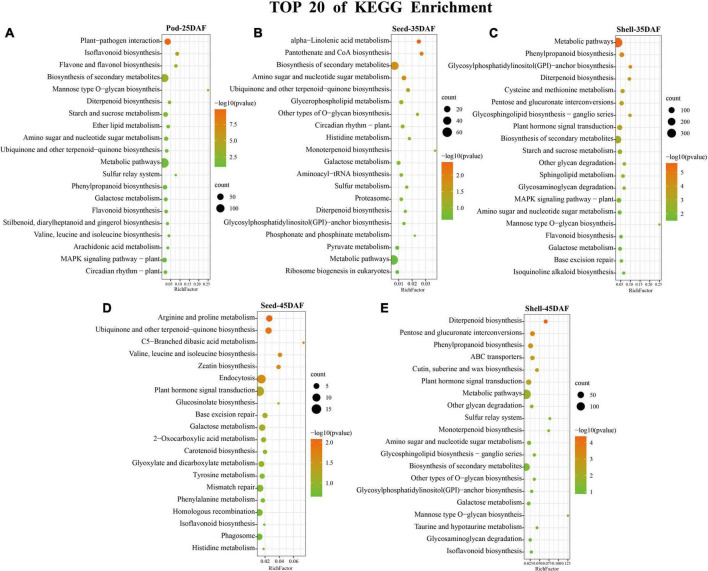
Kyoto Encyclopedia of Genes and Genomes (KEGG) Enrichment analysis. **(A–E)** Top 20 pathways of KEGG enrichment results in five phases: Pod-25, Seed-35, Shell-35, Seed-45, and Shell-45 DAF. The color scale indicates significance (corrected *p*-value). The size of the circle represents the number of genes enriched by that pathway.

In KEGG enrichment analysis, plant-pathogen interaction (ko04626), alpha-Linolenic acid metabolism (ko00592), metabolic pathways (ko01100), arginine and proline metabolism (ko00330), and diterpenoid biosynthesis (ko00904) pathways were most significantly enriched in Pod-25, Seed-35, Shell-35, Seed-45, and Shell-45 period, respectively (Figure 5 and Supplementary Table 5). Regarding the TOP 20 of KEGG enrichment, it is worth noting that DEGs were mainly enriched in metabolic pathways (ko01100), biosynthesis of secondary metabolites (ko01110), plant hormone signal transduction (ko04075) and MAPK signaling pathway–plant (ko04016). Metabolic pathways (ko01100) were significantly enriched in Shell-35, Shell-45, and Pod-25, plant hormone signal transduction (ko04075) pathways were significantly enriched in Shell-35 and Shell-45, and MAPK signaling pathway–plant (ko04016) was significantly enriched in Shell-35.

**FIGURE 6:**
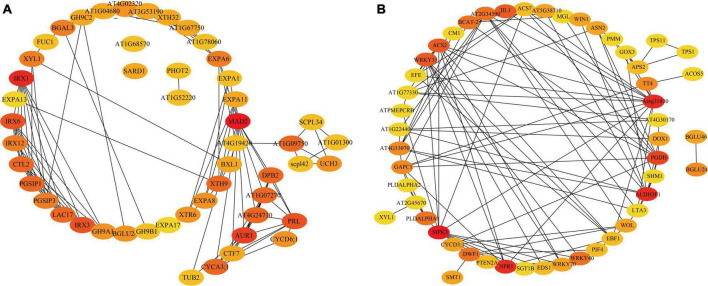
The DEG protein-protein interaction (PPI) network map. **(A)** The genes contained in the GO term of interest are plotted for PPI. **(B)** The genes contained in the KEGG pathway of interest were plotted for PPIs. The shade of the color represents the importance of interaction.

In addition, PPIs of DEGs were analyzed using the search tool for the retrieval of the interacting genes database. The DEGs identified within the GO terms of plant organ development, signal transduction, and hormone metabolism were subjected to PPIs analysis based on the Swissprot annotation results (Figure 6A). As a result, four hub proteins were identified, including IRX1 (Cellulose A synthase catalytic subunit 8), MAD2 (Mitotic spindle protein), AUR1 (Serine/threonine-kinase peak Aurora-1) and CYCA3; 1 (cyclin A3; 1). IRX1 is known to be involved in secondary cell wall biosynthesis (Taylor et al., 2000), MAD2 plays an important role in mitosis (Sikirzhytski et al., 2018), whereas AUR1 and CYCA3; 1 are involved in cell cycle regulations (Cao et al., 2017; Jean-Baptiste et al., 2019). The hub proteins revealed by PPIs analysis of the DEGs identified by the KEGG pathway (Figure 6B) include MPK3 (Mitogen-activated protein kinase 3), WRKY33, WRKY40, and PGDH (D-3-phosphoglycerate dehydrogenase/2-oxoglutarate reductase). MPK3 is known to be involved in the stress-mediated oxidative signaling cascade. WRKY40 participates in ABA-mediated seed germination and seedling development and acts as a TF in combination with an MPK3 promoter to regulate MPK3 expression (Wang T. J. et al., 2021; Boro et al., 2022). PGDH is involved in the phosphorylation pathway of serine biosynthesis which is an important link between primary metabolism and development (Toujani et al., 2013). Also, there were some enzymes that are related to cell growth and development, such as GAPC1 (Glyceraldehyde-3-phosphate dehydrogenase) and CYCD3; 1 (CYCLIN D3; 1), which are involved in the induction of mitotic cell division and play an important role in the switch from cell proliferation to the final stages of differentiation during plant development (Kim et al., 2013).

### Identification of differentially expressed transcription factors

**FIGURE 7:**
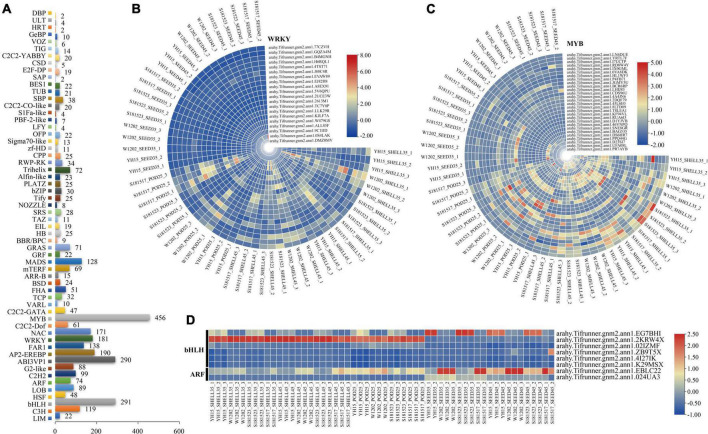
Identification of DEGs related to transcription factors (TFs). **(A)** Number of TFs expressed. **(B–D)** Heat map of relative expression of WRKY, MYB, bHLH, and ARF TF family DEGs. From red to blue, it indicates that the relative expression level is from high to low.

Transcription factors play an important role in the growth of different seed tissues by promoting or inhibiting cell division and expansion. Among the expressed genes, 3,366 genes encoding 58 families of TFs (Supplementary Table 6), of which 20 WRKY, 28 MYB, six bHLH, and two ARF of DEGs were found (Figure 7). Abundant *WRKY* and *MYB* DEGs were screened out in Pod-25 period, such as *arahy.2613M1* (WRKY), *arahy.5V6QPU* (WRKY), *arahy.A8EX91* (WRKY), *arahy.ALL85F* (WRKY), *arahy.46VNPQ* (MYB), *arahy.D1YJVB* (MYB), and all the WRKY family were down-regulated at this stage (Supplementary Tables 3, 6). Several *bHLH* and *MYB* DEGs were enriched in Shell-35, Shell-45, Seed-35, and Seed-45, such as *arahy.EG7BHI* (bHLH), *arahy.2KRW4X* (bHLH), *arahy.02IZMF* (bHLH), and *arahy.KF94VL* (MYB) (Supplementary Tables 3, 6).

### Identification of differentially expressed genes involved in phytohormones and mitogen-activated protein kinase signaling pathway

**FIGURE 8:**
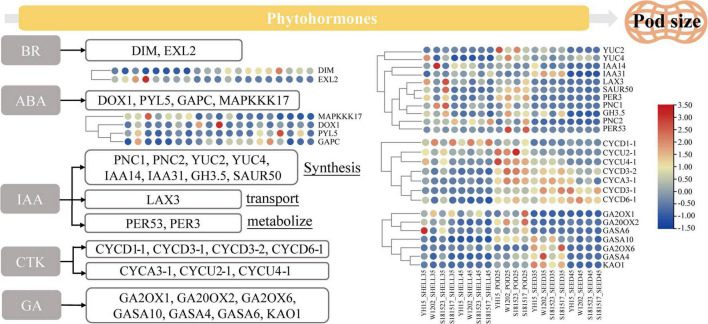
Heat map shows the relative expression of DEGs in BR, ABA, IAA, CTK, and GA phytohormone pathways. From red to blue, it indicates that the relative expression level is from high to low. The order of the sample IDs in the left figure is the same as in the right figure.

It has been recognized that the phytohormones, including auxin (such as IAA), brassinolide (BR), cytokinin (CTK), gibberellin (GA), and abscisic acid (ABA), are critical for pod size regulation (Li and Li, 2016; Li et al., 2019; Cao et al., 2020). Several GO terms and KEGG pathways were significantly enriched in multiple pod development phases as revealed by DEGs enrichment analysis, including gibberellin 20-oxidase activity (GO: 0045544), abscisic acid binding (GO: 0010427), and plant hormone signal transduction (ko04075), suggestive of imperative roles that phytohormones may play in regulating peanut pod development (Figure 8). In these pathways, numerous DEGs have been identified, which play critical roles in phytohormone regulation, including arahy.G65HTE (DIM) that is involved in the early synthesis of brassinolide (Liu et al., 2019), and arahy.U1WPAQ (EXL2) that plays a role in a brassinosteroid-dependent regulation of growth and development (Sun et al., 2019). ABA plays a dominant role in plant seed maturation and dormancy, arahy.ISVC9H (MAPKKK17) is a component of the ABA signaling pathway and is functionally related to the MAPK signaling pathway (Matsuoka et al., 2018), arahy.30RAY8 (DOX1) is involved in the negative regulation of ABA-mediated signaling pathways (Tirajoh, 2005), arahy.5CZ8DH (PYL5) is required for ABA-mediated responses (Gonzalez-Guzman et al., 2012), arahy.YH7XEC (GAPC) is a key enzyme in glycolysis (Guo et al., 2014). IAA promotes cell elongation and participates in regulating the structure and size of the embryo, as reflected by the enrichment of DEGs related to auxin synthesis, transport, and metabolism. For example, *arahy.VSM3NT* (*PNC1*), *arahy.2CFU61* (*PNC2*), *arahy.WRP4Q5* (*YUC2*), *arahy.6PM354* (*YUC4*), *arahy.5PTI62* (*IAA14*), and *arahy.IPD4BK* (*IAA31*), are related to auxin synthesis (Cheng et al., 2006; Guo et al., 2014; Butsayawarapat et al., 2019), *arahy.45C8U1* (*LAX3*) (Ng et al., 2015) are associated with transport, *arahy.RFF6WQ* (*PER53*), *arahy.ID0B6N* (*PER3*) are associated with auxin metabolism (Esmaeili et al., 2021). CTK promotes cell division and regulates plant growth and development, and plays an important role in seed development, as reflected by the highly expressed DEGs in the early stage of pod and seed developments, such as *arahy.RRC0V1* (*CYCU2-1*), *arahy.0ZFN4L* (*CYCU4-1*), and *arahy.69JDQK* (*CYCD3-2*). Furthermore, DEGs were also enriched to multiple *GASA* gene families that have been reported to play important roles in gibberellin-regulated pathways (Roxrud et al., 2007; Zhong et al., 2015), such as *arahy.F1TUFL* (*GASA4*), *arahy.VJ6NUA* (*GASA6*), and *arahy.V4B8PK* (*GASA10*). As aforementioned, *GASA* expression is regulated by BR, IAA, and ABA, indicative of the complexity and intertwined regulatory relationship of phytohormone related pathways in regulating peanut fruit development.

As a part of the MAPKKK-MAPKK-MAPK cascade, MAPKs play a crucial role in plant growth and development. In light of the KEGG enrichment analysis, the MAPK signaling pathway was significantly enriched in the Pod-25, Shell-35, and Seed-45 phases. A total of 65 DEGs were found to be relative to the MAPK pathway, of which arahy.RQ7QZ9 (MPK3) plays a redundant role with MAPK6 in biological function, arahy.ISVC9H (MAPKKK17), arahy.EJ82H8 (WRKY33), arahy.9C1IJD (WRKY40), and arahy.77CZVH (WRKY70) have been reported to interact with MPK3 and function in concert in the MAPK signaling pathway (Meng et al., 2012).

### Screening of candidate genes and SNP associated with pod size variation in YH15, W1202, S181523, and S181527

**FIGURE 9:**
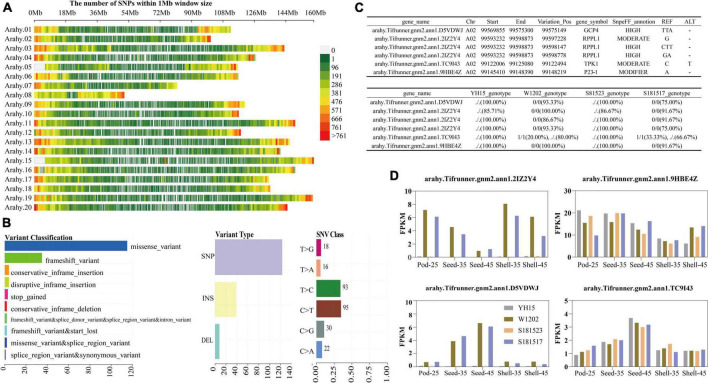
Variant loci associated with pod developmental traits were mined. **(A)** Genetic mapping of SNPs within 1Mb window size obtained by comparing them to the cultivated peanut reference genome. Chr01-chr20 corresponds to Arahy.01-Arahy.20. **(B)** Cohort summary plot displaying the distribution of variants according to variant classification, type, and SNV class. **(C)** Detailed information on the genetic variation of the four genes screened on the reported 92751772–99808878 bp QTL interval of the A02 chromosome.“./.” means identical to ALT genotype at the Variation_Pos and “0/0” means identical to REF genotype at the Variation_Pos. **(D)** FPKM values of *arahy.D5VDWJ*, *arahy.2IZ2Y4*, *arahy.TC9I43*, and *arahy.9HBE4Z* at different developmental stages.

In the transcriptome data derived from four accessions including YH15, W1202, S181523, and S181527, a total of 567,600 variant sites were acquired on the genome-wide scale after indexing the reference genome, two sequence alignments of the transcriptome sequencing data, and variant identification using GATK software, and 445,360 variant sites were obtained after filtering (Figure 9A). As shown by the horizontal histograms in Figure 9B, the top three most common mutation types were missense mutations, frameshift variation, and conservative inframe insertion. SNPs accounted for absolute positions compared with Indels, and C > T and T > C were the main mutation types (Figure 9B). In light of the recently available genome database of the cultivated peanut, a linkage genetic map was constructed by using the recombinant inbred population derived from a cross between Anthesis-36 and germplasm line 6-13 (Zhang S. et al., 2019). Seed weight (100SW), seed length (SL), seed width (SW), and length/width ratio (L/W) phenotypic data were collected in four conditions. Two stable QTL areas were identified on chromosomes 2 and 16 (positioned at 92,751,772–99,808,878 bp and 7,329,938–19,538,088 bp), harboring 514 and 684 candidate genes, respectively. A total of 35 variant sites were genotyped by transcriptome genetic variation information analysis in these two QTL regions, and 17 variant sites were found to reside on known genes by developed R scripts. On chromosome A02, four genes, including *arahy.D5VDWJ* (VariationPos: 99575149), *arahy.2IZ2Y4* (VariationPos: 99597228, 99598147, and 99598778), *arahy.TC9I43* (VariationPos: 99122494), and *arahy.9HBE4Z* (VariationPos: 99148219), which were annotated by the Swissprot database, have been reported to be relevant to plant growth and development (Figure 9C). Among them, the expression levels of *arahy.2IZ2Y4* and *arahy.D5VDWJ* in W1202 and S191517 were significantly higher than those of YH15 and S181523 across different pod developmental stages (Figure 9D), indicating their negative associations with pod size.

### Validation of differentially expressed genes from RNA-seq using quantitative real-time reverse transcriptase polymerase chain reaction

**FIGURE 10:**
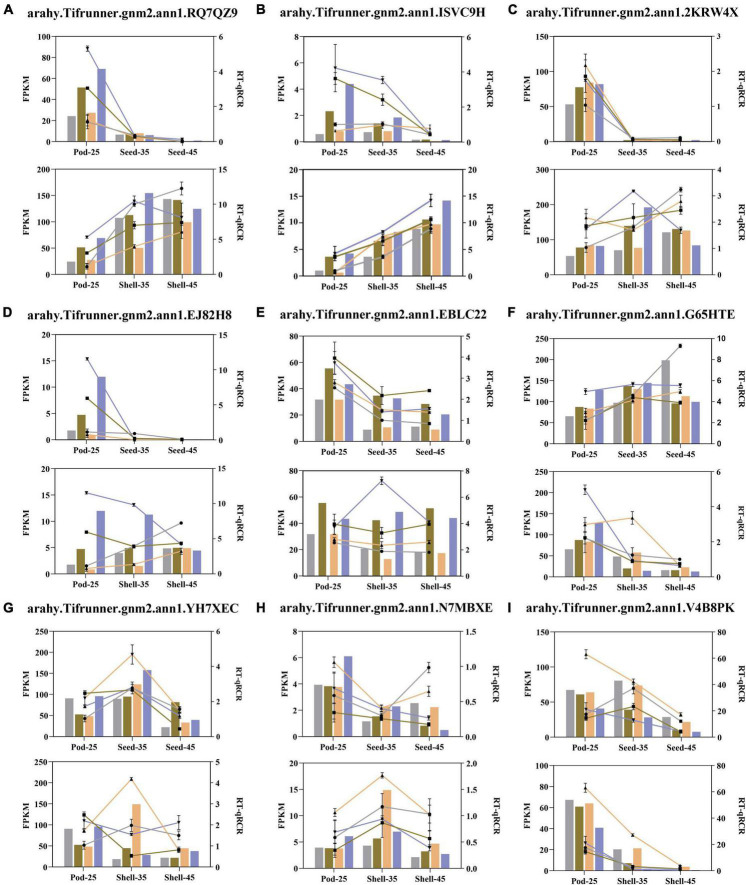
Quantitative real-time reverse transcriptase polymerase chain reaction (qRT-PCR) validation of 9 DEGs related to Pod size. **(A–I)** The horizontal coordinate attains the developmental period and the vertical coordinate represents the gene expression level. The representative colors of YH15, W1202, S181523, and S181527 are the same as in Figure 1. The line chart represents the relative expression determined with qRT-PCR and the column diagram represents the level of expression (FPKM) determined with RNA-seq. The relative expression levels were estimated from the threshold of the PCR cycle with the 2^–ΔΔ*Ct*^ method. Error bars indicate the standard errors from three independent biological and three technical replicates for qRT-PCR data.

In order to validate the expression patterns of DEGs obtained by RNA-Seq analysis, the expression levels of nine selected DEGs in the four varieties at five developmental stages were analyzed by qRT-PCR and displayed by two developmental types, including Pod-25 to Shell-45 and Pod-25 to Seed-45 (Figure 10 and Supplementary Table 7). These DEGs were selected from the enrichment results, including *arahy.RQ7QZ9* (*MPK3*), *arahy.ISVC9H* (*MAPKKK17*), *arahy.2KRW4X* (*MYC4*), *arahy.EJ82H8* (*WRKY33*), *arahy.EBLC22* (*ARF2a*), *arahy.G65HTE* (*DIM*), *arahy.YH7XEC* (*GAPC*), *arahy.N7MBXE* (*PYL5*), and *arahy.V4B8PK* (*GASA10*). Despite small variations in expression levels, the qRT-PCR results of almost all the nine genes showed expression patterns that are consistent with RNA-seq analysis, validating the results obtained by RNA-seq analysis.

## Discussion

Seed size is an important agronomic trait of crops, which plays a crucial role in determining seed yield (Sehgal et al., 2018; Lv et al., 2019). Dicotyledon seed development is primarily characterized by endosperm proliferation and embryonic expansion (Garcia et al., 2003). The embryo, endosperm, and the testa, which develop from the bead tepals, grow in concert and work together to determine the size of the seed (Bleckmann et al., 2014). The two cotyledons of the peanut absorb the endosperm, which is then replaced by the two swollen cotyledons, which serve as the major component of the edible. Despite being a major oilseed and cash crop worldwide, peanut has been seldomly subjected to pod size studies, at least with respect to the regulation mechanism of pod size, being lagged behind the grain crops such as rice, corn, and wheat, as well as legume crops such as soybean and alfalfa. This may, at least partially, be attributed to the peculiar technical difficulties of studying peanuts that are developed underground. In this study, RNA-seq was used to examine the transcriptional dynamics of four peanut lines with contrasting pod sizes at different developmental stages and to explore the molecular mechanisms that underpin pod development. In light of the gene expression data from five phases of pod (seed vs. shell) development, comparative studies on the transcriptional activities at different peanut pod development stages and the changes in gene expression patterns revealed an eclectic array of DEGs that are substantially regulated in concurrent with pod and seed development. In addition, several distinct transcriptional pathways that orchestrate seed development were unraveled by comprehensive transcriptome analysis coupled with variation site screening.

### Negative regulatory effects of WRKY and MYB on peanut pod size

**FIGURE 11:**
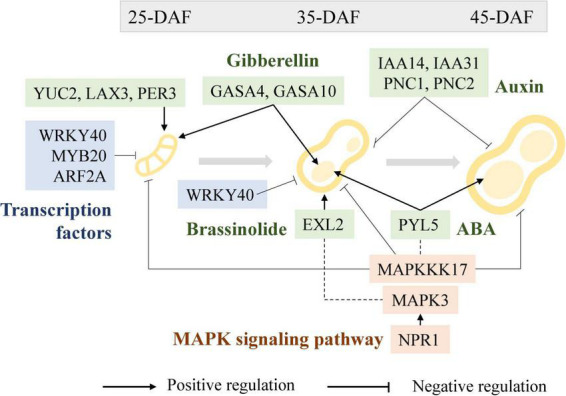
Regulatory roles of transcription factors (blue), phytohormones (green), and MAPK signaling pathway (pink) in peanut pod development.

Although environmental conditions influence plant seed development to some extent, genetic factors and their orchestrated gene expression during seed development is the primary determinant of seed size. TFs have a wide range of regulatory roles at the transcriptional level, many of which have been reported to play a crucial role in peanut pod growth and development as functionally verified in model plants or crops (Kaufmann et al., 2010; Sparks et al., 2013). *WRKY* is one of the largest transcriptional regulatory gene families in plants (Rushton et al., 2010), and it plays important role in abiotic stress responses and developmental processes such as embryogenesis and seed formation (Luo et al., 2005). For example, *GmWRKY15a* was identified to regulate seed size in wild soybean (Gu et al., 2017), and overexpression of *OsWRKY36* enlarged grain size in rice (Lan et al., 2020). Several DEGs in WRKY family transcription factors were highly expressed and regulated at the Pod-25, Shell-35, and Shell-45 periods. For example, the expression levels of *WRKY40* were significantly lower in the large pod genotypes YH15 and S181523 than in the small pod genotypes W1202 and S181517 at the Pod-25 and Shell-35 periods and expression levels increased concomitantly with the progress of shell and seed development, suggestive of a negative regulatory role in peanut pod development (Figure 11). It has been previously reported that in some legume crops, MYB and ARF are involved in seed size/weight determination (Gupta et al., 2017; Khemka et al., 2021). Dwindling in *MYB82*, *MYB3*, and *MYB44* expression was found to be concurrent with seed size enlargement in rapeseed seeds, corroborating evidence was also obtained in *MYB89*, overexpressing of which attenuated seed yield in Arabidopsis (Li et al., 2017; Wu et al., 2021). In agreement with these previous studies, we found that the expression of *MYB20* (*arahy.UFA08L* and *arahy.D23SI7*) exhibited gradual decline along with the peanut seed development over time, and also it showed relatively lower expression level in large-grain genotypes than in small-grain genotypes at a given developmental stage, suggesting a negative regulatory role during pod development stages. Likewise, ARF, which is known to be involved in auxin signal transduction, plays an important role in regulating the expression of auxin-responsive genes (Okushima et al., 2005; Van Ha et al., 2013; Chen et al., 2021). At a given developmental stage, the expression of *ARF2A* (*arahy.EBLC22*) that encodes an auxin signaling component implicated its potential role in fruit ripening and chloroplast formation (Breitel et al., 2016; Liu et al., 2021), was lower in large-grain genotypes than in small-grain genotypes.

### Phytohormone regulation in peanut pod growth and development

Multiple phytohormones could act in concert in a synergistic or antagonistic relationship in regulating pod and seed growth and development (Figure 8). For instance, low concentrations of the growth hormone indole-3-acetic acid promote peanut pod development (Moctezuma and Feldman, 1998; Moctezuma, 2003), and low concentrations of oleuropein lactone have a beneficial effect on the expansion of freshly incorporated fruit needles (Liu et al., 2020). Two ethylene release peaks at early pod expansion and at pod maturity, which are related to rapid expansion and regulation of pod maturation, respectively, are characteristics of peanut pods (Wang et al., 1992). Additionally, it was discovered that GA, along with CTK and ABA, positively regulated the accumulation of dry matter in the pod and seed kernel during pod enlargement (Luo et al., 2013). Physiological research on peanut pod development has shown that phytohormones have a crucial and vital regulatory role, but additional in-depth research on the molecular mechanisms of regulation is needed.

The expression of phytohormone-related genes, such as *EXL2*, was substantially upregulated in developing peanut shells relative to seeds, and gradually declined as peanut fruit developed over time. The expression of *EXL2* is required to suppress the brassinosteroid-dependent growth and control carbon partitioning in cells (Schröder et al., 2014). It is, therefore, conceivable that BR plays a role in shell growth, which is regulated by the expression of *EXL2* during pod development (Figure 11). Similarly, MAPK6 regulates BR response and cell proliferation, thereby affecting grain size in rice, and the functional redundancy between *MAPK6* and *MAPK3* suggests the presence of a link between the MAPK pathway and the BR response during rice grain growth and development (Tian et al., 2021).

Abscisic acid is another important phytohormone that is involved in regulating seed size and development (Tuan et al., 2018). As an ABA receptor, PYL regulates ABA-dependent gene expression, overexpression of which suppresses grain size in rice (Kim et al., 2014). In peanuts, we have observed progressively increased expression of *PYL5* concomitant to seed development in large-pod genotypes, which is incongruent with the previous report in rice. The impairment of *GAPC* led to the attenuation of the ABA signaling pathway (Muñoz-Bertomeu et al., 2011) and improved seed development by altering carbon flux in Arabidopsis (Zhang L. et al., 2019). During the Pod-25 period, *GAPC* expression was significantly lower in large-pod genotypes than in small-pod genotypes, suggesting its positive role in regulating pod size in peanuts. Furthermore, MAPKKK17 is engaged not only in the MAPK cascade but also in ABA-regulated leaf senescence in Arabidopsis (Jiao et al., 2017). At the shell-35, shell-45, Pod-25, and Seed-35 periods, *MAPKKK17* (*arahy.ISVC9H*) expression decreased along with pod development, with significantly lower expression in large-pod genotype than small-pod genotype at each developmental stage, showing a clear negative regulation on pod development (Figure 11).

PNC1 and PNC2 facilitate the reverse exchange of ATP and ADP or AMP in plant peroxisomes and play a key role in energy provision, the absence of which affects auxin metabolic processes (Linka et al., 2008; Nunes-Nesi et al., 2020). The expression levels of *PNC1* and *PNC2* were low in middle and late developmental seeds and significantly lower in large-grained than in small-grained at Pod-25, Shell-35, and Shell-45. The different expression patterns between seeds and shells suggest that *PNC1* (*arahy.VSM3NT*) and *PNC2* (*arahy.2CFU61*) expression played a greater role in the shell than that in the seed. *IAA14* (*arahy.5PTI62*), *IAA31* (*arahy.IPD4BK)*, auxin-responsive proteins, *SAUR50* (*arahy.8B51UP*), and auxin-responsive factor play negative regulation functions in auxin production and signal transduction (Bao et al., 2020). *IAA14 (arahy.5PTI62)* and *IAA31 (arahy.IPD4BK)* gene expression in the large-grained genotype was lower than that in the small-grained genotype, in the Shell-35 and Shell-45 period of shell development, but the difference was not conspicuous in other periods (Figure 11). *YUC2* encodes a flavin monooxygenase that catalyzes a rate-limiting step in auxin biosynthesis (Cheng et al., 2007; Ni et al., 2018). It was identified as a highly expressed gene in the large-pod genotypes at Pod-25. That expression of *LAX3* (*arahy.45C8U1*), which encodes an auxin influx protein and has positive feedback on auxin transport (Swarup et al., 2008), was significantly higher in large-grain genotypes than in small-grain genotypes during peanut fruit development. Moreover, auxin catabolism is mediated by the peroxidase superfamily proteins PER3 (arahy.ID0B6N) and PER53 (arahy.RFF6WQ), and their expression levels in large-pod genotypes are substantially higher than in small-pod genotypes at Pod-25 stage, while the opposite was true of the shells during pod development.

Cytokinin-related genes exhibited progressively elevated transcription levels in the pod development process, in particular, D-type cyclins (CYCD/CYCU) have been reported to play a prominent role in seed development by regulating the number of cells during seed development (Jameson and Song, 2016; Tuan et al., 2019). *CYCD1-1* (*arahy.KNXH3L*), *CYCD3-1* (*arahy.RN6XTC*), *CYCD3-2* (*arahy.69JDQK*), and *CYCD6-1* (*arahy.L9YRC6*) as D-type cyclins (CYCD) are overtly differentially expressed concurrently with peanut fruit development. Interestingly, the cell area of the seed increased and was more closely aligned with each other from 35 DAF to 45 DAF. Observing the longitudinal-section of the seed, the cell count of large-grain was significantly higher than that of small-grain peanuts. The above CYCD/CYCU gene family genes were expressed in higher amounts in the early and middle stages, and much higher than in the middle and late shells, which is consistent with the result that cytokinins regulate cell numbers during seed development (Figure 2). Notably, *AUR1* and *IRX1* occupy an important position in the PPI map. Knockdown mutants of *AUR1* cause major defects in lateral root formation and growth, regulating the direction of the formative division plane orientation during development. On the other hand, plant cell growth involves both cell expansion and cell wall reinforcement, and the cell growth pattern is largely dependent on cell wall cellulose synthesis and alignment (Wang et al., 2019). *IRX1* appears in several cell biogenesis-related GO terms, and it catalyzes cellulose synthesis in cell walls implying that the regulation of cell expansion and cell wall reinforcement is related to pod development (Xu et al., 2013). Their potential role in regulating pod/seed size in peanuts is intriguing and warrants further investigation.

Gibberellic acid stimulated Arabidopsis (*GASA*) expression is regulated by GA and known to be involved in plant hormone signal transduction that affects seed size (Fan et al., 2017; Trapalis et al., 2017; Ahmad et al., 2020). In Arabidopsis, the seed size was smaller in *GASA4* defective mutant but significantly larger in the *GASA4*-overexpressing lines than in WT (Roxrud et al., 2007). Furthermore, *GASA10* encodes a cell wall protein, the overexpression of which results in shorter siliques and fewer seeds (Trapalis et al., 2017). It has been suggested that *GASA10* is involved in silique formation by controlling the number of hydroxyl radicals in certain areas of the cell wall that governs cell wall elongation. In this study, *GASA4* (*arahy.F1TUFL*) and *GASA10* (*arahy.VJ6NUA*) exhibited substantially higher gene expression in the large-pod genotypes at Pod-25 and Seed-35 than in small-pod genotypes, suggestive of their positive regulatory roles in pod development in promoting pod enlargement (Figure 11). GA promotes cell elongation and play a significant role in promoting the accumulation of dry matter in seed during peanut seed expansion, and section observations also revealed a gradual accumulation of intracellular material. *GASA4* and *GASA10* were not only differentially expressed between materials in Pod-25 and Seed-35, but were also at peak expression, which corresponded perfectly to the production of peak release during early pod expansion. An eclectic range of phytohormones, such as BR, IAA, ABA, and GA, were all involved in regulating the expression of the *GASA* gene family, which gives an inkling that GAGA may act a central role in modulating phytohormone signal transduction networks that are critically important in pod/seed size determination.

### Mitogen-activated protein kinase signaling pathway and pod growth and development

Mitogen-activated protein kinase signaling pathways play an essential role in rice grain size regulation (Guo et al., 2018; Xu et al., 2018b; Zhang and Zhang, 2022). In this study, the MAPK pathway-related genes, including *MPK3* (*arahy.RQ7QZ9*), *WRKY33* (*arahy.EJ82H8*), *WRKY40* (*arahy.9C1IJD*), and *NPR1* (*arahy.KS67M3*) were identified as key DEGs derived by KEGG enrichment, suggesting that they may occupy an important place in the protein-protein interaction networks that govern peanut pod/seed development. It has been reported that MPK3 modulates fiber initiation and elongation in cotton (Wang N. N. et al., 2021), root apical meristem mitotic activity in Arabidopsis (Shao et al., 2020), and bead-coat cell division in Arabidopsis (Wang et al., 2008). The expression level of *MPK3* was significantly lower in the large-pod genotypes than in the small-pod genotypes in pod-25 stage. Interestingly, further down the track of fruit development, it was expressed significantly higher in the shells than in the seeds, as indicated by the DEG profiles of shell-35 vs. seed-35 and shell-45 vs. seed-45 (Supplementary Figure 8). *NPR1* is necessary for ubiquitinated substrates of E3 ligase complexes (Zavaliev et al., 2020), and is involved in promoting MPK3 and MPK6 activation in the MAPK pathway (Yi et al., 2015; Backer et al., 2019). It is worthy of note that *NPR1* showed a consistent pattern with that of *MAPK3* during peanut fruit development, with substantially higher expression in the shell than in the seeds.

### Variation site mining

Variant site mining has seldomly been conducted in prior transcriptome investigations, which can not only explore the molecular mechanisms that underpin the traits of interest by virtue of the expression profiles of the candidate genes but also help to narrow down or even pinpoint the key genes by leveraging the segregation of relevant phenotypes among the genotypes with similar genetic backgrounds. In the A02 chromosomal interval relevant to seed size, four genes were found to have stable genotyping, among which *GCP4* (*arahy.D5VDWJ*) exhibited a lower expression level in the large-pod peanuts than in small-pod peanuts. *GCP4* is an important component of ɣ-tubulin in microtubule nucleation in plant cells, transgenic overexpression of which gives rise to dwarf phenotypes and smaller organs (Kong et al., 2010; Ma et al., 2021), which is well in line with our observation that higher *GCP4* expression in small-pod peanuts than in large-pod peanuts. It is interesting to note that the expressions of *RPPL1* (*arahy.2IZ2Y4*) and *GCP4* (*arahy.D5VDWJ*) differed significantly between large-pod and small-pod peanut genotypes.

Given that these genes are implicated in resistance to Fusarium wilt and downy mildew in plants (Bittner-Eddy et al., 2000; Shao et al., 2019), are potential disease resistance genes. It is intriguing to investigate their roles in plant defense against the pathogen in the association of pod size in future studies. Taken together, the validation of these findings will need to screen for molecular markers in broader populations and identify prospective genes/markers using genome-wide association analysis, which are anticipated to facilitate the genetic improvements of the cultivated peanuts for desirable seed size and optimal seed yields through molecular breeding.

## Data availability statement

The data can be accessed through the following link: https://www.ncbi.nlm.nih.gov/bioproject/?term=PRJNA847769 or through the SRA accession number: PRJNA847769.

## Author contributions

YW, ZS, and XZ conceived the study and drafted the manuscript. YW, FQ, WD, BH, and ZZ discussed the writing plan. MT, JW, RZ, XWa, and XWu performed the experiments. XS and HL obtained the experimental materials. YW analyzed the data. All authors have read, reviewed, and approved the submitted version.

## Footnotes

1
https://www.fao.org/faostat/zh/#home

2
https://www.bioinformatics.babraham.ac.uk/projects/fastqc/

3
https://peanutbase.org/data/v2/Arachis/hypogaea/genomes/

4
https://www.omicshare.com/

5
https://github.com/broadinstitute/picard

6
http://planttfdb.gao-lab.org/

7
https://www.r-project.org

8
https://www.ncbi.nlm.nih.gov/tools/primer-blast/
