# Supplementary material for: Comparative transcriptomics analysis of developing peanut (Arachis hypogaea L.) pods reveals candidate genes affecting peanut seed size
Source: Front Plant Sci. 2022 Sep 12;13:958808. doi: 10.3389/fpls.2022.958808 (PMC9511224; doi:10.3389/fpls.2022.958808)
Supplement: Supplementary file 2 [file Data_Sheet_2.docx]

**Supplementary Figure 1**: Significant comparison of cell area and cell size in seeds and shells. (A) Shell cell area in cross-section, S181523 was significantly higher at 35 DAF than at 45 DAF. (B) Number of seed cells in longitudinal-section, S181523 was significantly more numerous than S181517 at 45 DAF. (C) Shell cell area in longitudinal-section, YH15 was significantly higher at 35 DAF than at 45 DAF.

**
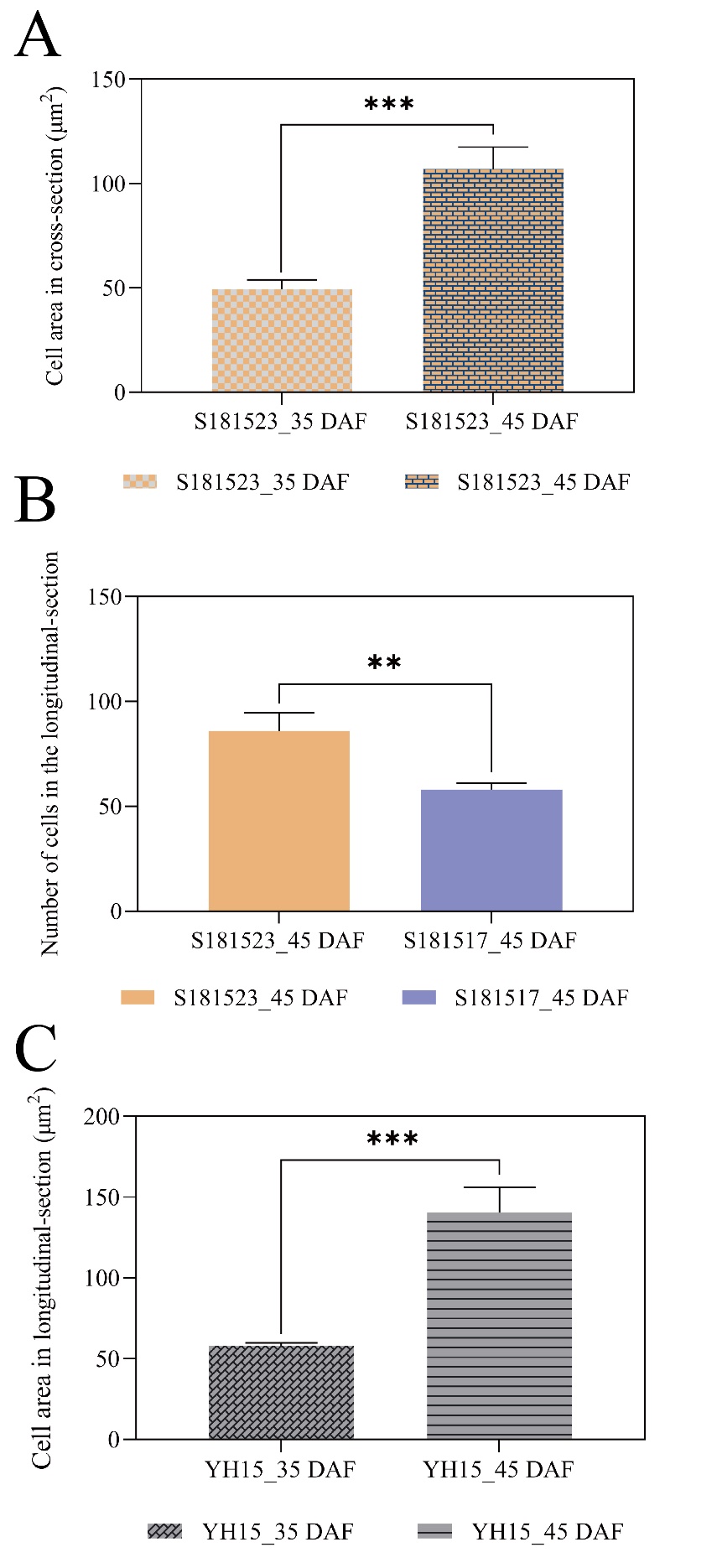
**

**Supplementary Figure 2**: Number of gene expression between materials at different developmental stages.

**
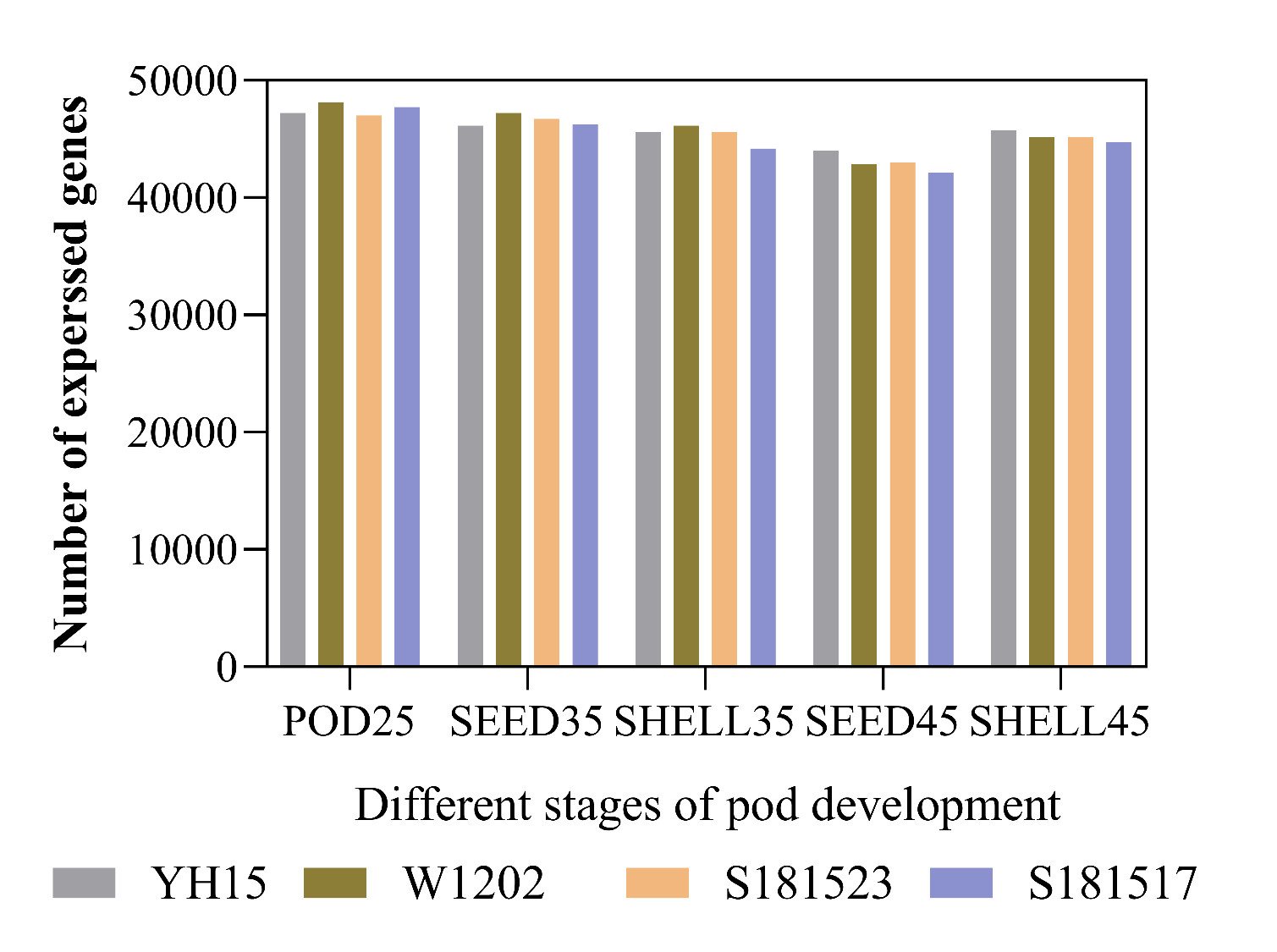
**

**Supplementary Figure 3**: Principal component analysis (PCA).

**
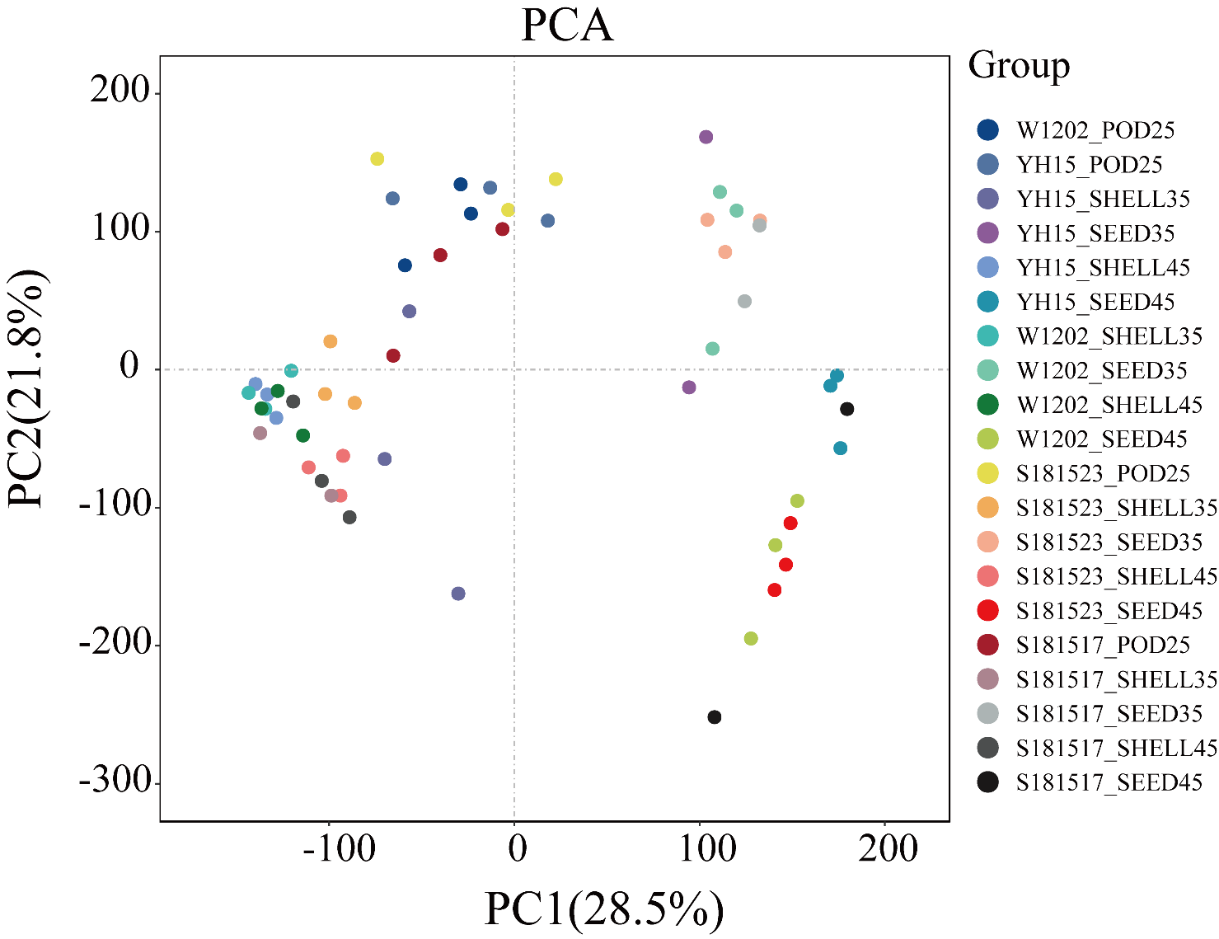
**

**Supplementary Figure 4**: Correlation analysis between samples.

**
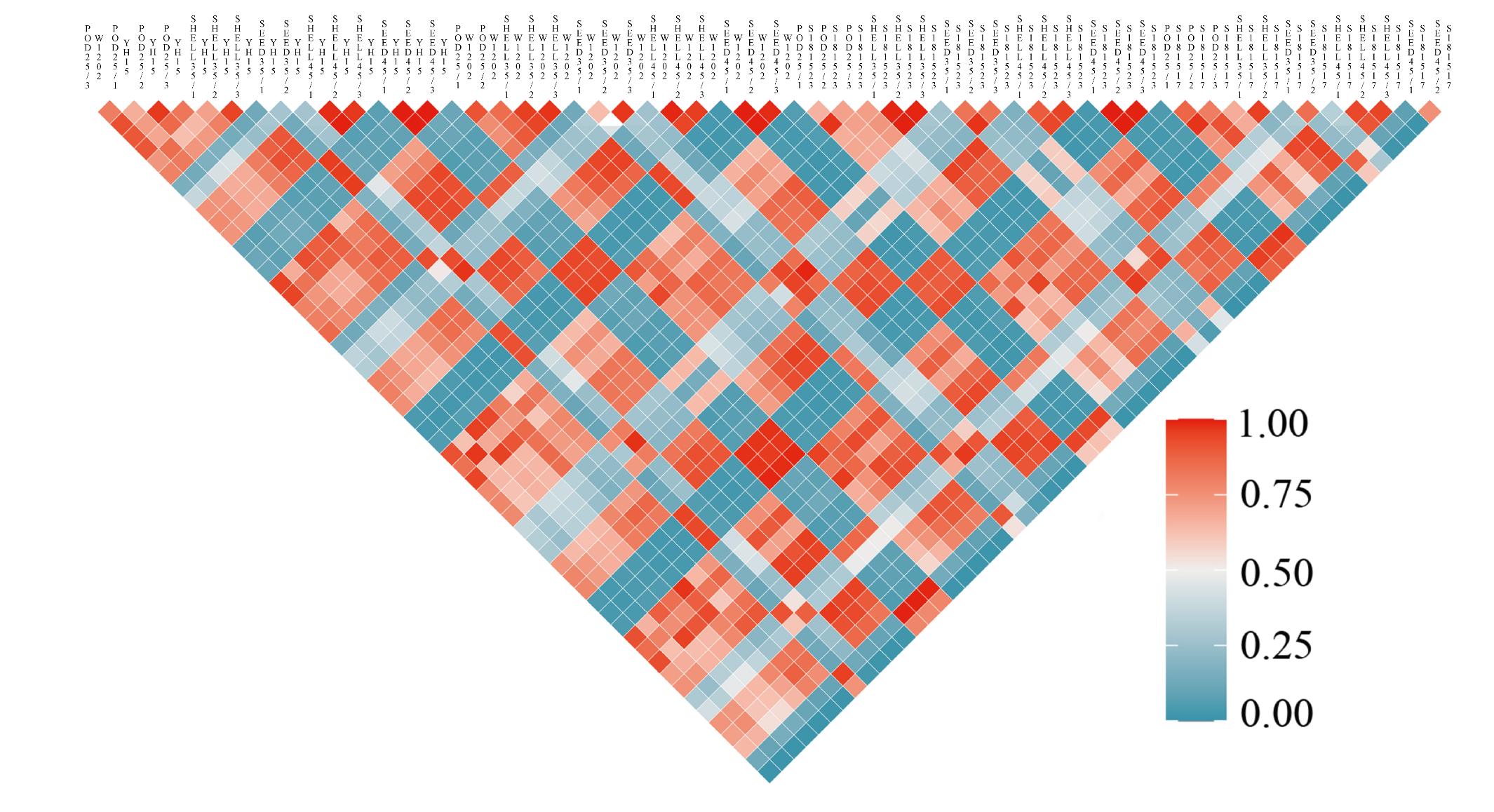
**

**Supplementary Figure 5**: Overall expression distribution of 56 peanut pod transcriptome sequencing samples.


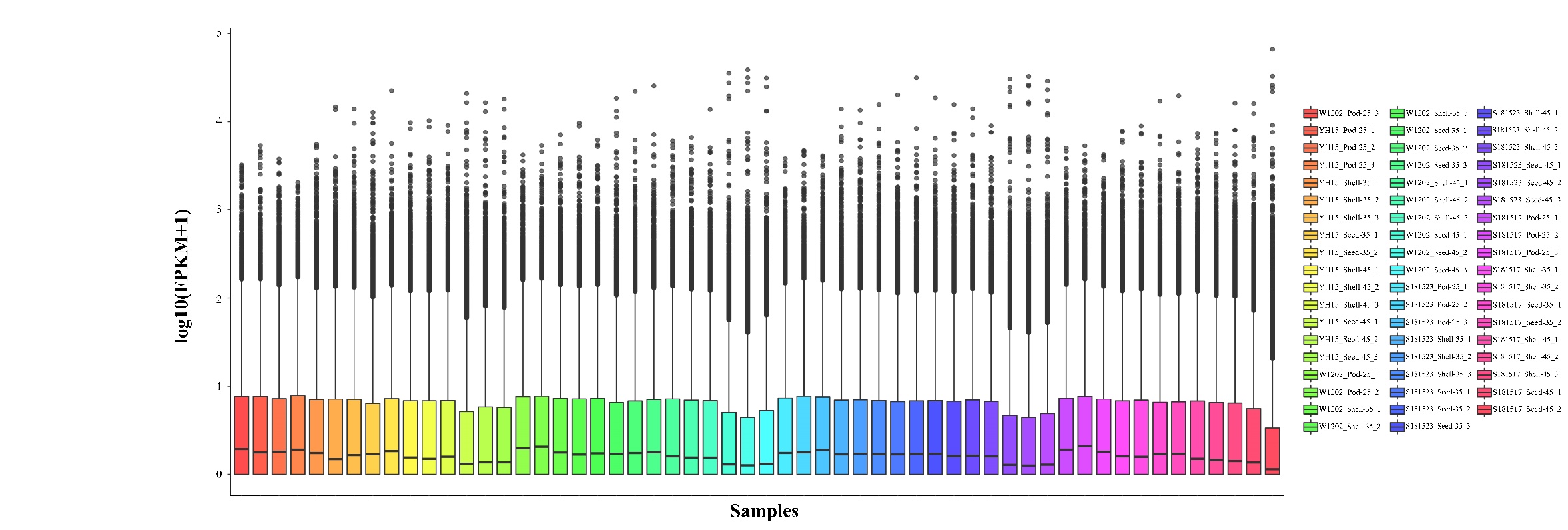


**Supplementary Figure 6**: Nr, Swissprot, Eggnog, GO and KEGG database annotation results.

**
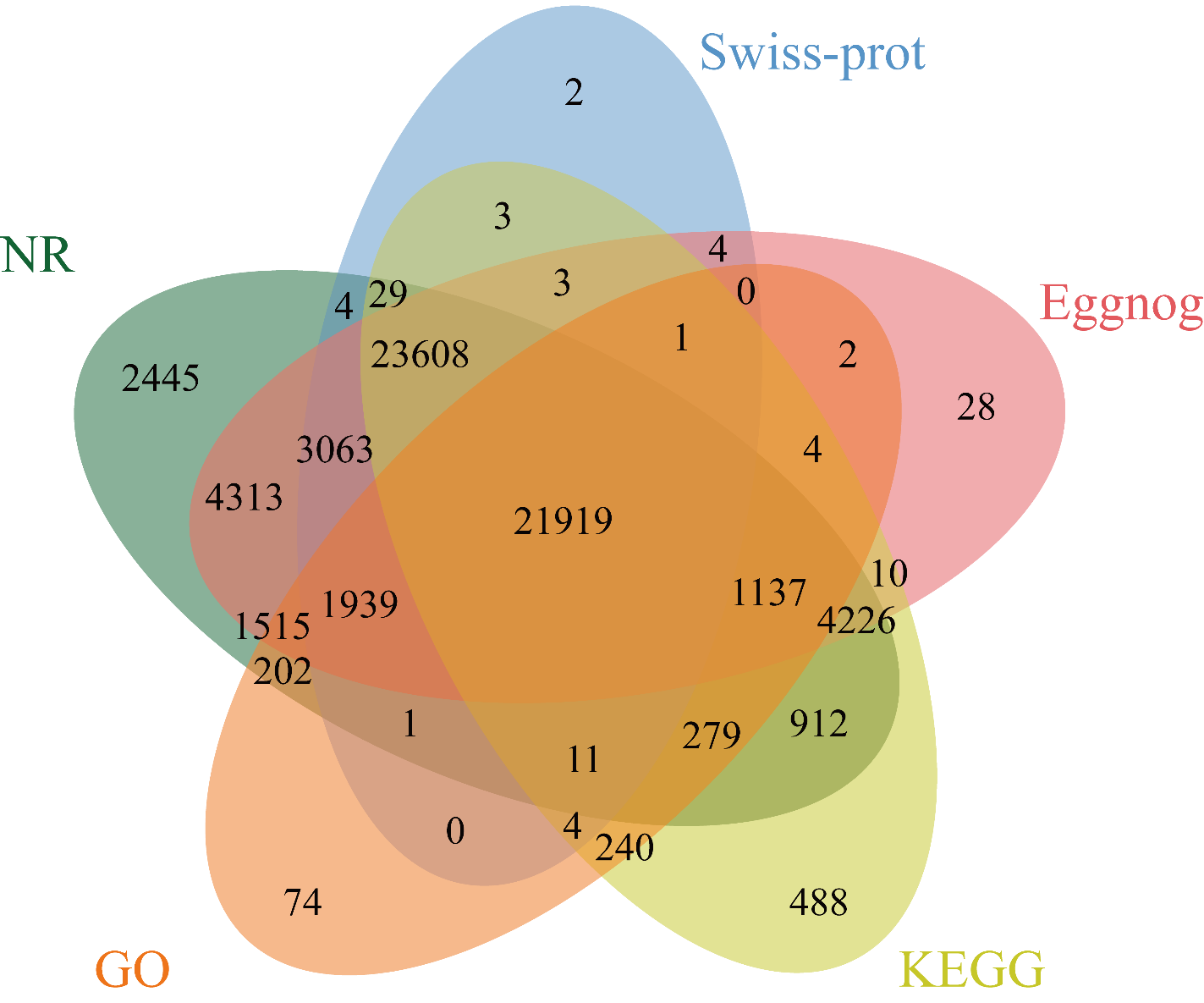
**

**Supplementary Figure 7**: flower plot of DEG analysis at five developmental stages from four accessions, YH15 vs W1202 and S181523 vs S181527.

**
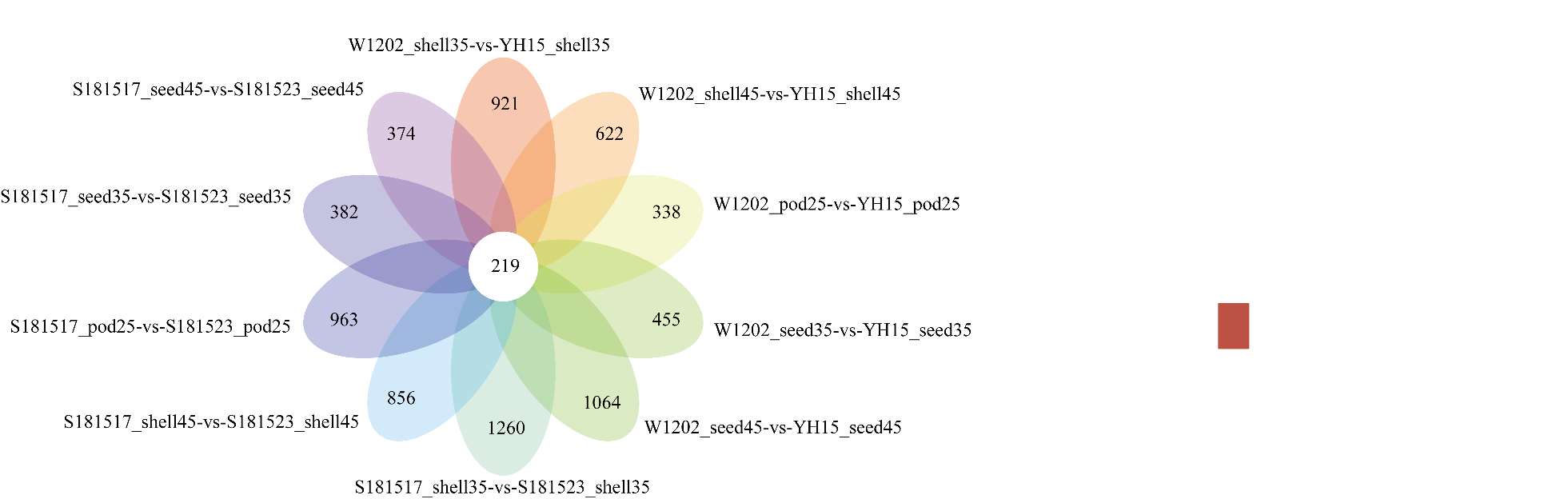
**

**Supplementary Figure 8**: Heat map shows the relative expression of DEGs in MAPK signaling pathways. From red to blue, it indicates that the relative expression level is from high to low.

**
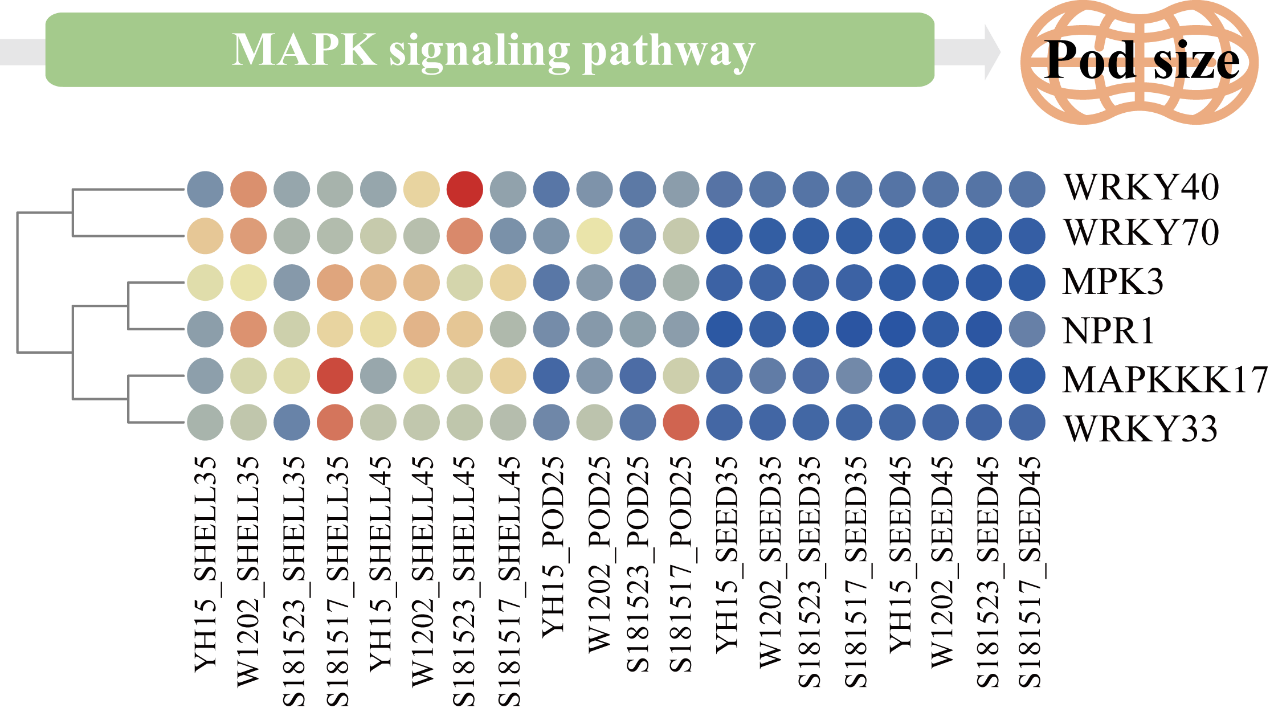
**
